# Introduction of Human Flt3-L and GM-CSF into Humanized Mice Enhances the Reconstitution and Maturation of Myeloid Dendritic Cells and the Development of Foxp3^+^CD4^+^ T Cells

**DOI:** 10.3389/fimmu.2018.01042

**Published:** 2018-05-28

**Authors:** Ryutaro Iwabuchi, Shota Ikeno, Mie Kobayashi-Ishihara, Haruko Takeyama, Manabu Ato, Yasuko Tsunetsugu-Yokota, Kazutaka Terahara

**Affiliations:** ^1^Department of Immunology, National Institute of Infectious Diseases, Tokyo, Japan; ^2^Department of Life Science and Medical Bioscience, Waseda University, Tokyo, Japan; ^3^Department of Mycobacteriology, Leprosy Research Center, National Institute of Infectious Diseases, Tokyo, Japan; ^4^Department of Medical Technology, School of Human Sciences, Tokyo University of Technology, Tokyo, Japan

**Keywords:** humanized mice, dendritic cells, cytokines, Flt3-L, GM-CSF, T cells, Foxp3

## Abstract

Two cytokines, fms-related tyrosine kinase 3 ligand (Flt3-L) and granulocyte-macrophage colony-stimulating factor (GM-CSF) are considered to be the essential regulators of dendritic cell (DC) development *in vivo*. However, the combined effect of Flt3-L and GM-CSF on human DCs has not been evaluated *in vivo*. In this study, we, therefore, aimed at evaluating this using a humanized mouse model. Humanized non-obese diabetic/SCID/Jak3^null^ (hNOJ) mice were constructed by transplanting hematopoietic stem cells from human umbilical cord blood into newborn NOJ mice, and *in vivo* transfection (IVT) was performed by hydrodynamic injection-mediated gene delivery using plasmids encoding human Flt3-L and GM-CSF. Following IVT, Flt3-L and GM-CSF were successfully induced in hNOJ mice. At 10 days post-IVT, we found, in the spleen, that treatment with both Flt3-L and GM-CSF enhanced the reconstitution of two myeloid DC subsets, CD14^−^CD1c^+^ conventional DCs (cDCs) and CD14^−^CD141^+^ cDCs, in addition to CD14^+^ monocyte-like cells expressing CD1c and/or CD141. GM-CSF alone had less effect on the reconstitution of these myeloid cell populations. By contrast, none of the cytokine treatments enhanced CD123^+^ plasmacytoid DC (pDC) reconstitution. Regardless of the reconstitution levels, three cell populations (CD1c^+^ myeloid cells, CD141^+^ myeloid cells, and pDCs) could be matured by treatment with cytokines, in terms of upregulation of CD40, CD80, CD86, and CD184/CXCR4 and downregulation of CD195/CCR5. In particular, GM-CSF contributed to upregulation of CD80 in all these cell populations. Interestingly, we further observed that Foxp3^+^ cells within splenic CD4^+^ T cells were significantly increased in the presence of GM-CSF. Foxp3^+^ T cells could be subdivided into two subpopulations, CD45RA^−^Foxp3^hi^ and CD45RA^−^Foxp3^lo^ T cells. Whereas CD45RA^−^Foxp3^hi^ T cells were increased only after treatment with GM-CSF alone, CD45RA^−^Foxp3^lo^ T cells were increased only after treatment with both Flt3-L and GM-CSF. Treatment with Flt3-L alone had no effect on the number of Foxp3^+^ T cells. The correlation analysis demonstrated that the development of these Foxp3^+^ subpopulations was associated with the maturation status of DC(-like) cells. Taken together, this study provides a platform for studying the *in vivo* effect of Flt3-L and GM-CSF on human DCs and regulatory T cells.

## Introduction

Dendritic cells (DCs) play a pivotal role in maintaining the immune responses (1, 2). DCs comprise multiple subsets with distinct functions but can be broadly classified into two major subsets, myeloid DCs [including classical/conventional DCs (cDCs), monocyte-derived DCs (MoDCs), and Langerhans cells] and plasmacytoid DCs (pDCs), on the basis of ontogeny (3–11). Two cytokines, fms-related tyrosine kinase 3 ligand (Flt3-L) and granulocyte-macrophage colony-stimulating factor (GM-CSF), are considered to be the essential regulators of DC development *in vivo*: Flt3-L supports the development of cDCs and pDCs derived from bone marrow (BM) progenitors, while GM-CSF contributes to the development of MoDCs as well as inflammation-induced myeloid DCs (3, 4, 10, 12, 13). One study using knock-out mice showed that combined deficiency of Flt3-L and GM-CSF, rather than a single deficiency of either cytokine, massively reduced not only DCs in the periphery but also monocyte-macrophage DC progenitors and further downstream common DC progenitors in the BM, indicating the concerted action of Flt3-L and GM-CSF on DC homeostasis *in vivo* (13). Cytokines, such as IL-3, IL-4, IL-15, TNF-α, and TGF-β are selectively responsible for the development and maturation of specific DC subsets, which affects the type of immune response that ultimately develops (6, 7). However, in humans, the effect of Flt3-L and GM-CSF singly or in combination in the absence of any other cytokine on the development of DCs remains to be evaluated *in vivo*.

Humanized mice, which are reconstituted with human immune cells, provide an opportunity to study human hematopoiesis *in vivo*. Recent advances in the development of humanized mice have been achieved by using second-generation immunodeficient mouse strains, such as non-obese diabetic (NOD)/SCID/IL2Rγ^null^ (NSG or NOG), NOD/Rag1^null^/IL2Rγ^null^ (NRG), and BALB/c/Rag2^null^/IL2Rγ^null^ (BRG) mice, in all of which the IL-2 receptor common γ-chain is defective, preventing host B, T, and NK cell development, allowing for efficient xenotransplantation (14–16). In addition, xenotransplantation of human hematopoietic stem cells (HSCs) instead of human peripheral blood mononuclear cells into immunodeficient mice enables long-term and multi-lineage human hematopoiesis (17–21). Our research group has also developed a humanized mouse model using human HSC-transplanted NOD/SCID/Jak3^null^ (NOJ) mice (22–24). NOJ mice, which have an identical phenotype as NSG and NOG mice due to the deficiency of IL-2 downstream molecule Jak3, were developed as an alternative recipient mouse strain for humanization (25). However, some issues with these humanized mouse models still need to be overcome. One of these issues is the limited biologic cross-reactivity of cytokines between mice and humans, which leads not only to insufficient development of human hematopoietic cells, especially myeloid-lineage cells, but also to insufficient human innate and adaptive immunity (14, 15, 26, 27). To overcome this, several approaches for introducing human cytokines have been proposed, including the development of genetically engineered mouse strains, administration of recombinant proteins, and hydrodynamic injection of cytokine gene-encoding plasmids (14, 16, 27). For example, treatment of humanized NSG mice with human GM-CSF and IL-4 by hydrodynamic injection of plasmids successfully enhances DC reconstitution and induced antigen-specific immune responses (28). In addition, humanized NSG-SGM3 mice, into which human stem cell factor, GM-CSF, and IL-3 genes are genetically introduced, display increased human myeloid cells (specifically myeloid DCs) (29). However, it should be noticed that additional cytokines can also affect other immune cell populations, including T cells, and non-physiological exposure to cytokines during T cell development can influence the cell populations that are generated (16, 29). Indeed, human GM-CSF and IL-4-introduced humanized NSG mice accelerated the maturation/activation of CD4^+^ T cells (28), and humanized NSG-SGM3 mice displayed skewed development of Foxp3^+^ regulatory T cells (Tregs) within the CD4^+^ T cell population (29).

In this study, we aimed at evaluating the effect of Flt3-L and GM-CSF singly or in combination in the absence of any other cytokine on the reconstitution and maturation of human DCs *in vivo* using a humanized mouse model. Our humanized NOJ (hNOJ) mice were rather beneficial than other genetically engineered humanized mouse models, in terms of evaluating the effect of exogenous human cytokines. In order to exogenously introduce human Flt3-L and GM-CSF into hNOJ mice, we used the hydrodynamic gene delivery technique, since this is a simple and efficient method to express cytokines in mice (28, 30, 31). The reconstitution and maturation of systemic human DC subsets in hNOJ mice were evaluated following expression of these cytokines *in vivo*. T cell populations were also evaluated in terms of the induction of Foxp3^+^ Treg(-like) cells and the development status (naïve/memory) of other T cell subsets.

## Materials and Methods

### Construction of hNOJ Mice

Human HSCs were isolated from umbilical cord blood using the CD133 MicroBead Kit (Miltenyi Biotec, Tokyo, Japan). Humanized NOJ mice were constructed as described previously (23, 25), with minor modifications. In brief, freshly isolated human HSCs (1–1.5 × 10^5^ cells) were transplanted into the livers of non-irradiated NOJ mice (≤2 days old). Approximately 20 µl of peripheral blood was periodically obtained from the facial vein to determine the extent of chimerism [the percentage of human CD45 (hCD45)^+^ cells within total peripheral blood cells]. The individual mice used in this study are listed in Table S1 in Supplementary Material with information on chimerism and the HSC donor ID number. It should be noted that the development of T cells is delayed compared with that of myeloid cells and B cells, and at least 12 weeks is required to see substantial development of T cells in the periphery after transplantation of HSCs into hNOJ mice (23), as in other humanized mouse models (17, 21). Therefore, 15- to 17-week-old hNOJ mice (old mice) were generally used, except for in the experiments, in which 4-week-old hNOJ mice (young mice) were used. All mice were maintained under specific pathogen-free conditions in the animal facility at the National Institute of Infectious Diseases (NIID).

### IVT of Human Flt3-L and GM-CSF by Hydrodynamic Gene Delivery in hNOJ Mice

The open reading frames for the genes encoding human Flt3-L and GM-CSF (GenBank: NM_001459.3 and NM_000758.3, respectively) were subcloned separately into the pEF-BOS-bsr plasmid (32). Plasmid DNA was purified using the NucleoBond Xtra Maxi EF Kit (Macherey-Nagel, Düren, Germany). For hydrodynamic gene delivery, hNOJ mice were intravenously injected with 50 µg of each plasmid in TransIT-QR Hydrodynamic Delivery Solution (Mirus, Madison, WI, USA) within 4 s using a 27-gauge needle. As a control, 50 µg of the empty vector pEF-BOS-bsr plasmid was administered.

### Measurement of Plasma Cytokines

For this analysis, peripheral blood was collected from the tail vein of hNOJ mice. Human Flt3-L and GM-CSF in the plasma of hNOJ mice was determined by cytometric bead array (CBA) (33). Plasma samples were serially diluted with the Assay Diluent supplied in the BD CBA Human Soluble Protein Master Buffer Kit (BD Biosciences, San Diego, CA, USA). The Human GM-CSF Flex Set (BD Biosciences) was used to measure circulating human GM-CSF. To measure circulating human Flt3-L, an anti-human Flt3-L monoclonal capture antibody (40416; R&D Systems, Minneapolis, MN, USA) was conjugated to beads (Functional Bead A9; BD Biosciences) that have a distinct fluorescent intensity from the human GM-CSF capture bead using the Functional Bead Conjugation Buffer Set (BD Biosciences). A biotinylated anti-human Flt3-L polyclonal antibody (R&D Systems) was used as a detection antibody and was treated with phycoerythrin (PE)-conjugated streptavidin (BioLegend, San Diego, CA, USA). Data were collected on a FACSCanto II (BD Biosciences) and analyzed using FCAP Array Software v3.0 (Soft Flow Inc., St. Louis Park, MN, USA) based on the fluorescent intensity of PE. Standard curves were set using recombinant human Flt3-L (R&D Systems) and GM-CSF (supplied in the Human GM-CSF Flex Set). The limit of detection of both human Flt3-L and GM-CSF was <0.01 ng/ml.

### Cell Preparation

Cells were prepared from the peripheral blood, spleen, and BM of hNOJ mice. The peripheral blood was collected from the tail vein before the initiation of IVT and was treated with EDTA-2Na at a final concentration of 5 mM. Splenocytes were prepared at 10 days post-IVT using the Spleen Dissociation Kit mouse (Miltenyi Biotec) and the gentleMACS Dissociator (Miltenyi Biotec). BM cells were prepared by flushing the femurs and tibias of naïve hNOJ mice and hNOJ mice at 10 days post-IVT. Peripheral blood and cells isolated from the spleen or BM were treated with ACK buffer (0.15 M NH_4_Cl, 1 mM KHCO_3_, and 0.1 mM EDTA-2Na; pH 7.2−7.4) for 3 and 1 min, respectively, at RT to lyse the red blood cells and then suspended in staining buffer (PBS containing 2% fetal bovine serum and 0.01% sodium azide). For myeloid cell phenotyping by flow cytometry, cells were suspended in PBS containing 0.5% bovine serum albumin and 5 mM EDTA-2Na.

### Flow Cytometry for Human Leukocytes

The fluorochrome-conjugated monoclonal antibodies used are listed in Table 1. All monoclonal antibodies except one that is specific for mouse CD45 (mCD45) were specific for human antigens. An FcR Blocking Reagent (Miltenyi Biotec) was used to prevent non-specific binding of monoclonal antibodies. The Live/Dead Fixable Dead Cell Stain Kit (ThermoFisher Scientific, Waltham, MA, USA) was used for staining dead cells, which were gated out during analysis. All of the cells collected from the peripheral blood samples and 0.5–1 × 10^6^ cells from the BM and spleen were stained with the mixture of fluorochrome-conjugated antibodies, FcR blocking reagent, and the Live/Dead reagent in staining buffer for 30 min on ice. Intracellular staining for Foxp3 was performed for 1 h on ice using the Foxp3 Staining Buffer Set (eBioscience/ThermoFisher Scientific) after cell surface staining. Data from all cells stained were collected on a FACSCanto II or FACSAria III (BD Biosciences). For determining the absolute cell numbers, 20 µl of peripheral blood or 1/1,000 of the cells obtained from the BM and spleen were stained with antibodies specific for human CD45 (hCD45) and mCD45 and the Live/Dead reagent in Trucount tubes (BD Biosciences) for 30 min at RT. After staining, cells were resuspended in buffer (ACK buffer for peripheral blood samples, and staining buffer for BM and spleen samples), and the cells were subjected to flow cytometry without washing. Data were collected on a FACSCanto II until 3 × 10^4^ of the reference beads in the Trucount tubes were acquired. All data were analyzed using FACSDiva software (BD Biosciences) or FlowJo software (LLC, Ashland, OR, USA). The absolute numbers of each cell population were calculated based on the percentages within live CD45^+^ cells.

**Table 1:** Monoclonal antibodies used for flow cytometry.

Name	Clone	Conjugate	Source
CD1c	L161	Alexa Fluor 700	BioLegend
CD3	HIT3a	APC^c^	BioLegend
CD3	UCHT1	Brilliant Violet 605	BioLegend
CD4	RPA-T4	PerCP^d^	BioLegend
CD4	OKT4	Brilliant Violet 605	BioLegend
CD8a	RPA-T8	PerCP	BioLegend
CD11b	ICRF44	FITC^e^	BioLegend
CD11c	Bu15	PE	BioLegend
CD14	RMO52	ECD	Beckman Coulter^i^
CD19	HIB19	Brilliant Violet 605	BioLegend
CD19	HIB19	APC	eBioscience^j^
CD27	O323	FITC	BioLegend
CD33	P67.6	APC-Cy7^f^	BioLegend
CD34	581	FITC	BioLegend
CD38	HIT2	PerCP	BioLegend
CD40	5C3	FITC	BioLegend
hCD45^a^	HI30	Pacific Blue	BioLegend
mCD45^b^	30-F11	FITC	BioLegend
CD45RA	HI100	PE-Cy7^g^, Brilliant Violet 785	BioLegend
CD56	5.1H11	Brilliant Violet 605	BioLegend
CD80	2D10	PE	BioLegend
CD86	IT2.2	PerCP-Cy5.5^h^	BioLegend
CD116/GM-CSFR	4H1	PE	BioLegend
CD123	6H6	PE-Cy7	BioLegend
CD135/Flt3	4G8	Alexa Fluor 647	BD Biosciences
CD141	M80	Brilliant Violet 785	BioLegend
CD184/CXCR4	12G5	PE	BioLegend
CD195/CCR5	HEK/1/85a	FITC	BioLegend
CD303	AC144	FITC	Miltenyi Biotec
Foxp3	259D	PE	BioLegend
HLA-DR	L243	PerCP	BioLegend
Isotype control			
Mouse IgG1	MOPC-21	FITC, PE, PerCP	BioLegend
Mouse IgG1	MOPC-21	Alexa Fluor 647	BD Biosciences
Mouse IgG2a	7T4-1F5	ECD	Beckman Coulter
Mouse IgG2a	MOPC-173	PE, PerCP	BioLegend
Mouse IgG2b	MPC-11	PerCP-Cy5.5	BioLegend
Rat IgG2a	RTK2758	FITC	BioLegend

*^a^Human CD45*.

*^b^Mouse CD45*.

*^c^Allophycocyanin*.

*^d^Peridinin–chlorophyll protein*.

*^e^Fluorescein isothiocyanate*.

*^f^Allophycocyanin-cyanin 7*.

*^g^Phycoerythrin-cyanin 7*.

*^h^Phycoerythrin-cyanin 5.5*.

*^i^Brea, CA, USA*.

*^j^San Diego, CA, USA*.

### Microscopic Analysis

Cells isolated on a FACSAria III were cytospun onto glass slides and stained with the May–Grünwald Stain Solution (Wako Pure Chemical Industries, Osaka, Japan) and Giemsa Stain Solution (Wako Pure Chemical Industries). Slides were visualized on a CKX41 microscope (Olympus, Tokyo, Japan), and images were captured with a Macromax camera (Goko Camera, Kanagawa, Japan).

### Statistical Analysis

*T*-tests (unpaired, parametric) and Mann–Whitney *U*-tests (unpaired, non-parametric) were used for two-group comparisons. For multiple comparisons, data were analyzed by one-way ANOVA (unpaired, parametric) followed by Holm–Sidak’s test (parametric), or by Kruskal–Wallis test (unpaired, non-parametric) followed by Dunn’s multiple comparisons test (non-parametric). The Spearman’s rank correlation coefficient (non-parametric) was used for correlation analyses. The variance of the data being compared was determined by F-test for two-group comparisons and by the Brown–Forsythe test for multiple comparisons. When the variance was significant, a non-parametric test was used. GraphPad Prism version 6 (GraphPad Software, CA, USA) was used for all analyses. A *P*-value of <0.05 was considered statistically significant.

## Results

### Successful Induction of Human Flt3-L and GM-CSF by IVT

**Figure 1:**
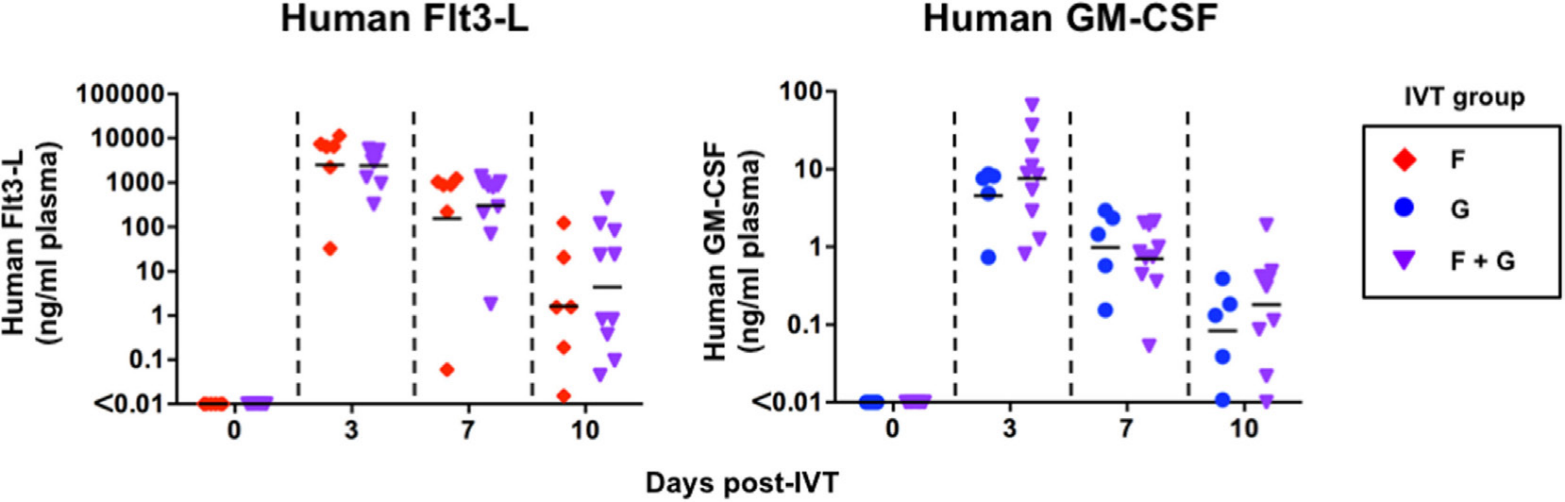
Induction of human fms-related tyrosine kinase 3 ligand (Flt3-L) and granulocyte-macrophage colony-stimulating factor (GM-CSF) in hNOJ mice following in vivo transfection (IVT). Humanized NOJ mice were injected with plasmids expressing human Flt3-L (Group F), GM-CSF (Group G), or both (Group F + G). Plasma samples obtained at the indicated time points were subjected to cytometric bead array. Data are the individual values and the geometric mean for each group (Group F: *n* = 6, Group G: *n* = 5, Group F + G: *n* = 10). The Mann–Whitney *U* test was used to compare IVT groups, and no significant differences were observed at any time point (*P* > 0.05).

Humanized NOJ mice at the steady state (day 0) showed undetectable levels of human Flt3-L and GM-CSF in the plasma (<0.01 ng/ml; Figure 1). When hNOJ mice were injected with the Flt3-L-expressing plasmid (Group F), the GM-CSF-expressing plasmid (Group G), or both plasmids (Group F + G), the corresponding cytokines could be induced within 3 days of IVT. The mean concentrations of plasma Flt3-L at day 3 post-IVT were 2,533 and 2,762 ng/ml in Group F and Group F + G, respectively, and those of plasma GM-CSF were 4.5 and 7.6 ng/ml in Group G and Group F + G, respectively. The levels of these cytokines gradually decreased with time, but were detectable for at least 10 days post-IVT. No significant differences in the concentration of either Flt3-L or GM-CSF between the IVT groups were observed at any time. These results indicate that IVT by hydrodynamic injection-mediated gene delivery is a useful method to transiently introduce human cytokines into hNOJ mice.

### Characterization of Putative DC Populations in hNOJ Mice at the Steady State

**Figure 2:**
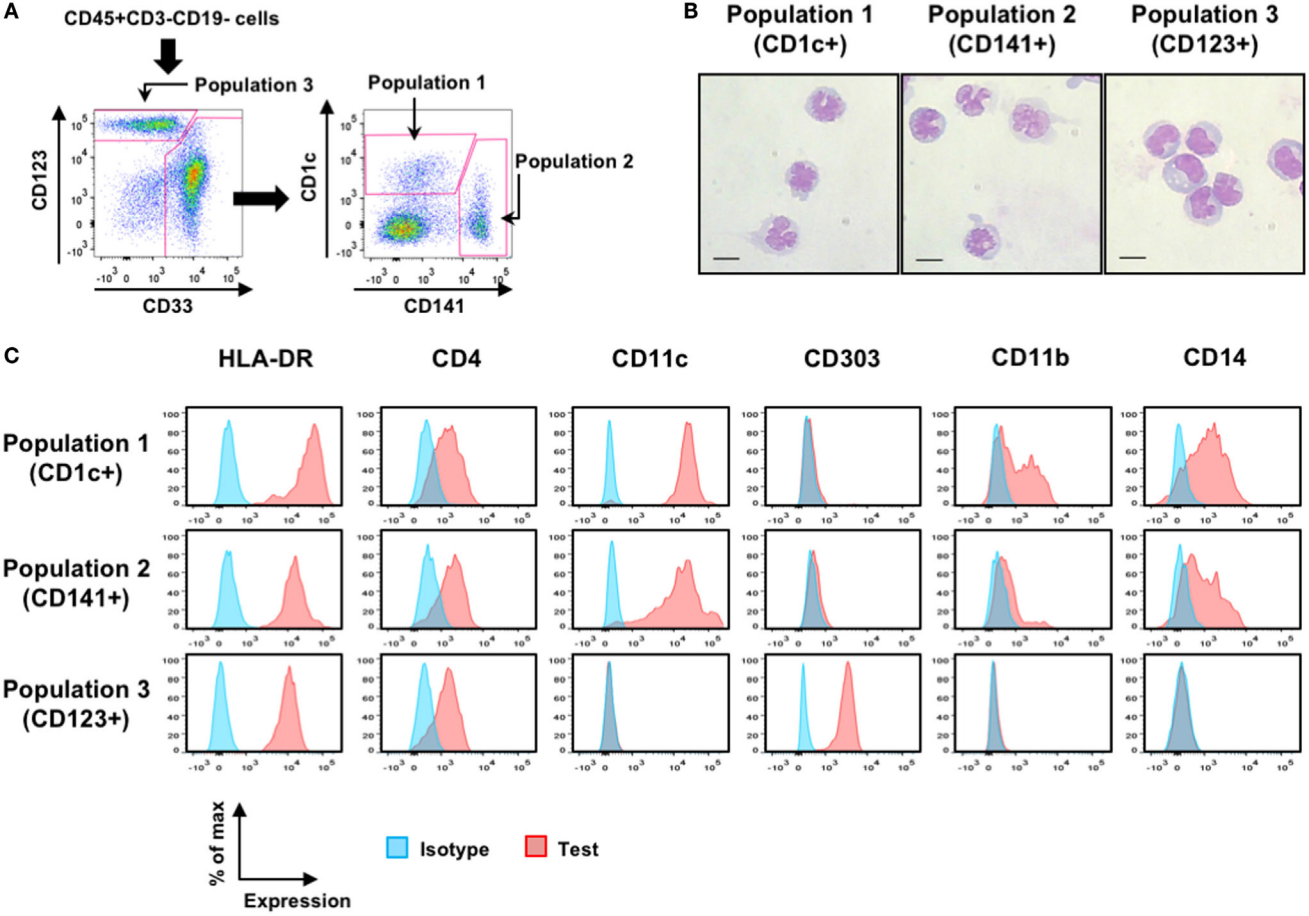
Characterization of putative human dendritic cell (DC) populations in hNOJ mice. **(A)** A representative gating strategy for flow cytometry of CD1c^+^ population (Population 1), CD141^+^ population (Population 2), and CD123^+^ population (Population 3) using bone marrow (BM) cells from Group E. **(B)** May–Grünwald and Giemsa staining of each sorted putative DC population using pooled BM from four naïve hNOJ mice. Scale bars are 10 µm. **(C)** Representative histograms for subset-associated markers on splenic populations from Group E.

Human DCs are well characterized on the basis of cell surface markers including CD1c (for cDC2), CD141 (for cDC1), and CD123 (for pDC) (7, 8, 11, 34–36). However, some monocyte-like DC subsets such as “inflammatory” DCs can express CD14 (8, 34). Therefore, although we used cell surface markers of CD1c, CD141, and CD123 to distinguish each putative DC population, we did not deplete CD14^+^ cells during cell preparation. Cells at the steady state were prepared from the BM and spleen specimens from naïve hNOJ mice or hNOJ mice that were injected with the empty vector pEF-BOS-bsr plasmid (these mice are referred to as Group E hereafter). To distinguish putative DC populations, CD45^+^CD3^−^CD19^−^ cells were divided into CD123^+^CD33^+/−^ population (Population 3) and CD123^+/−^CD33^+^ myeloid cells. CD123^+/−^CD33^+^ myeloid cells were subdivided into CD1c^+^ population (Population 1) and CD141^+^ population (Population 2) (Figure 2A). May–Grünwald and Giemsa staining revealed the typical morphologies for each DC subset (20, 35): Population 1 and Population 2 exhibited a similar morphology to cDCs, with a less round shape and multilobulated nuclei, whereas Population 3 exhibited a similar morphology to pDCs, with a round shape and indented nuclei (Figure 2B). We next analyzed the expression profiles of subset-associated markers (HLA-DR, CD11c, CD303, CD4, CD11b, and CD14) (Figure 2C). All three putative DC populations expressed HLA-DR, a defining feature of antigen-presenting cells (37). As with all of DC subsets in humans (7, 35, 36, 38), all three populations in hNOJ mice expressed CD4. CD11c and CD303 are used as distinctive markers for human cDCs and pDCs, respectively (7–9, 11, 35, 36), and myeloid cell populations (Population 1 and Population 2) and Population 3 in hNOJ mice could clearly be distinguished by these markers. Population 1 and Population 2, but not Population 3, in hNOJ mice expressed CD11b and CD14, though the expression of CD11b was more evident in Population 1 than in Population 2. These results indicate that Population 3 in hNOJ mice was phenotypically identical to pDCs in human blood. By contrast, Population 1 and Population 2 were heterogeneous, consisting of CD14^−^ genuine cDCs and CD14^+^ monocyte-like cells.

### Assessment of CD14^+^ Monocyte-Like Cells in Putative DC Populations Following Cytokine Induction

**Figure 3:**
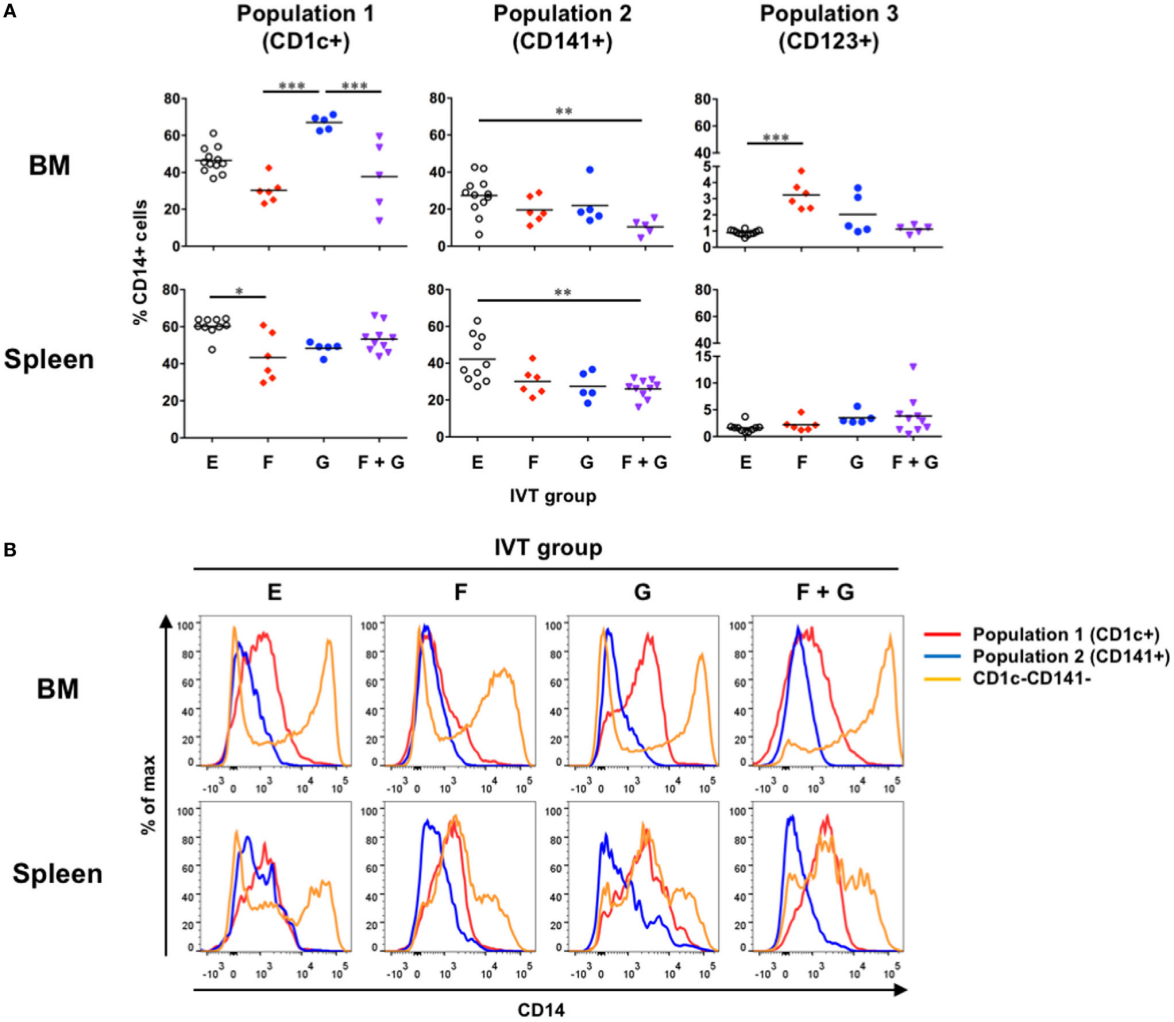
Development of CD14^+^ monocyte-like cells in hNOJ mice. Cells were prepared from the bone marrow and spleen of hNOJ mice within each *in vivo* transfection (IVT) group. **(A)** The percentages of CD14^+^ cells within CD1c^+^ population (Population 1), CD141^+^ population (Population 2), and CD123^+^ population (Population 3) were compared across the IVT groups (*n* = 5–12 per group). Significant differences (*P < 0.05, **P < 0.01, ***P < 0.001) were determined by the Kruskal–Wallis test followed by the Dunn’s multiple comparisons test. **(B)** Representative histograms of CD14 expression among Population 1, Population 2, and CD1c^−^CD141^−^ myeloid cells.

We next asked how much CD14^+^ monocyte-like cells were included in each putative DC population in hNOJ mice following cytokine induction. On flow cytometry, it should be noted that CD1c and CD141 double-positive cells were often observed within myeloid cell compartments, especially in the presence of Flt3-L (Figure S1 in Supplementary Material), and these cells were counted as CD141^+^ cells, as described elsewhere (39). An obvious finding was that CD14^+^ monocyte-like cells were significantly enriched in Population 1 in the BM after treatment with GM-CSF alone (Figure 3A, upper left). Although Population 3 in the BM also involved increased CD14^+^ monocyte-like cells when Flt3-L was induced, the percentage of CD14^+^ monocyte-like cells was minimal (<5%) (Figure 3A, upper right). Apart from these cases, the ratios of CD14^+^ monocyte-like cells in every cytokine-induced IVT group were similar or decreased compared with those at the steady state in the BM and spleen (Group E). We further compared the level of CD14 expression among Population 1, Population 2, and CD1c^−^CD141^−^ myeloid cells. Notably, whereas CD1c^−^CD141^−^ myeloid cells included cells expressing higher level of CD14, the level of CD14 expression in Population 1 and Population 2 in the BM and spleen was low or intermediate in any cytokine-induced IVT group (Figure 3B). Since three types (classical, intermediate, and non-classical) of monocytes are defined in human blood and CD14 expression level of classical and intermediate monocytes is higher than that of non-classical monocytes (40), CD14^+^ monocyte-like cells within Population 1 and Population 2 would be separated from classical and intermediate monocytes.

### Enhanced Reconstitution of DCs Following Cytokine Induction

**Figure 4:**
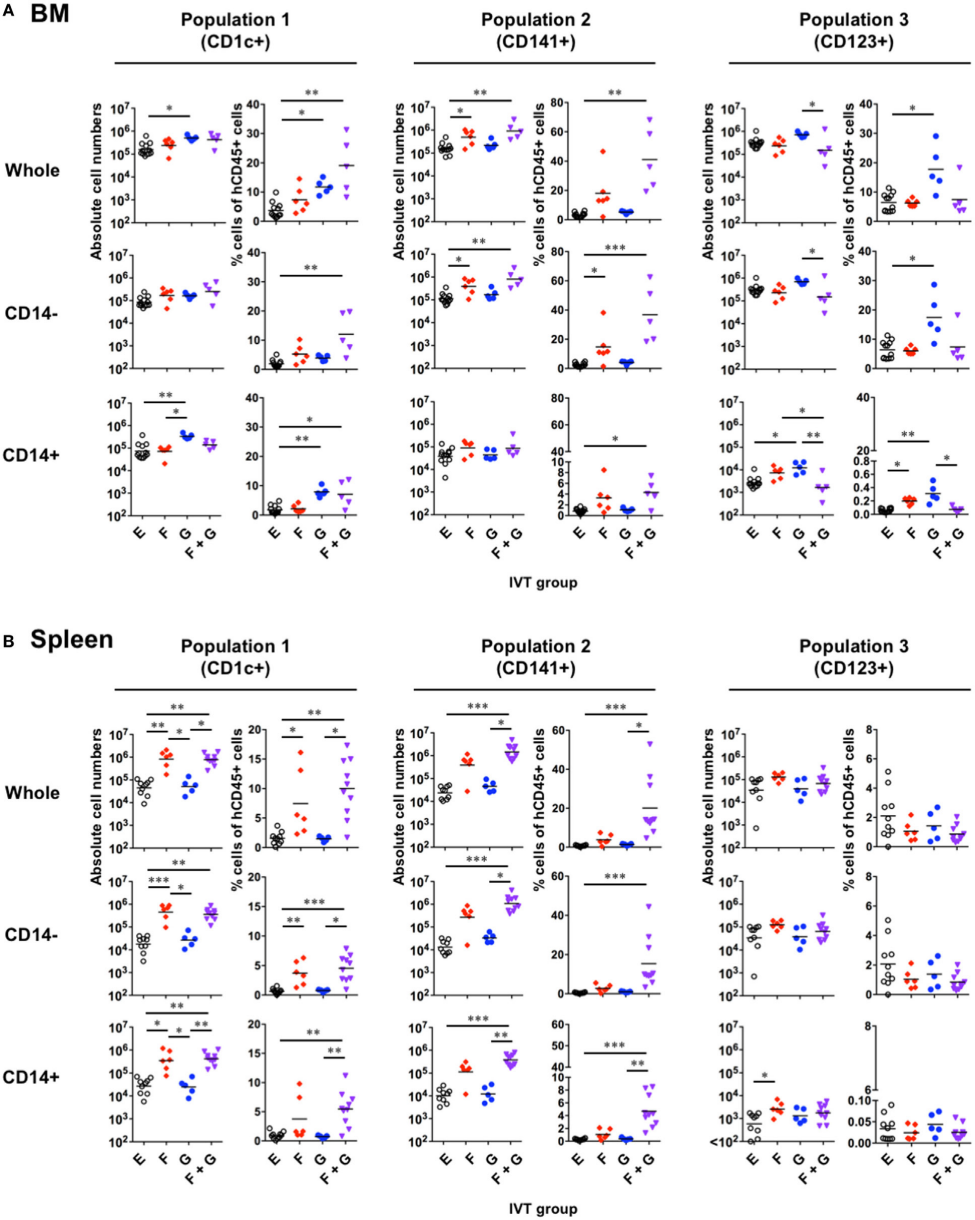
Reconstitution of putative human dendritic cell populations in hNOJ mice following *in vivo* transfection (IVT). Cells were prepared from the bone marrow (BM) and spleen of each IVT group. **(A,B)** Comparison of the absolute cell numbers (left panels) and the percentages (right panels) of CD1c^+^ population (Population 1), CD141^+^ population (Population 2), and CD123^+^ population (Population 3) among all hCD45^+^ cells in the BM **(A)** and spleen **(B)**. Data are the individual values with the geometric means of the absolute cell numbers and the means of the percentages (*n* = 5–12 per group). Significant differences (*P < 0.05, **P < 0.01, ***P < 0.001) were determined by the Kruskal–Wallis test followed by the Dunn’s multiple comparisons test.

Given that CD14^+^ monocyte-like cells substantially existed in any IVT group (Figure 3), each putative DC population was subdivided into CD14^−^ DCs and CD14^+^ monocyte-like cells to assess the effect of Flt3-L and GM-CSF on the reconstitution of each cell population in hNOJ mice. The absolute cell numbers and the percentages of each cell population were measured in the BM and spleen, and the reconstitution levels were compared among the IVT groups (Figure 4). This percentage would be informative if there was individual variability in the reconstitution levels of human leukocytes in the BM and spleen prior to IVT; however, chimerism, as determined by the percentage of hCD45^+^ cells in the peripheral blood population at the initiation of IVT, was not significantly different among the IVT groups (Figure S2 in Supplementary Material).

In the BM (Figure 4A), GM-CSF seemed to be responsible for the increased reconstitution of Population 1 and, surprisingly, Population 3, but not Population 2. In other words, the absolute cell numbers and/or the percentages of Population 1 and Population 3 were increased in the presence of GM-CSF. However, the increased reconstitution of Population 1 after treatment with GM-CSF alone could be attributed to that of CD14^+^ monocyte-like cells, but not CD14^−^CD1c^+^ cDCs. Indeed, CD14^+^ monocyte-like cells were significantly enriched in Population 1 after treatment with GM-CSF alone (Figure 3A). On the other hand, Population 3 after treatment with GM-CSF alone involved the increased reconstitution of both CD14^−^ pDCs and CD14^+^ monocyte-like cells, though the contamination of CD14^+^ monocyte-like cells into Population 3 was a negligible level (<4%) (Figure 3A). In contrast to GM-CSF, Flt3-L was required for the increased reconstitution of Population 2, and this could be substantially attributed to that of CD14^−^CD141^+^ cDCs. Interestingly, whereas an additive effect of Flt3-L and GM-CSF was observed on the reconstitution of both CD14^−^ cDCs and CD14^+^ monocyte-like cells in Population 1 and Population 2, this was not true for pDC (Population 3) reconstitution.

The reconstitution profiles in the spleen (Figure 4B) differed from what was observed in the BM, likely due to egression/immigration and/or the site-specific milieu responsible for cell maintenance. An obvious difference between the two organs was observed with respect to Population 1: Flt3-L increased the reconstitution of this population including both CD14^−^ cDCs and CD14^+^ monocyte-like cells in the spleen, but not the BM. In addition, few pDCs (Population 3) were expanded in the spleen in any IVT group.

### Assessment of the *In Vivo* Effect of Flt3-L on the Reconstitution of pDCs Using Young hNOJ Mice

**Figure 5:**
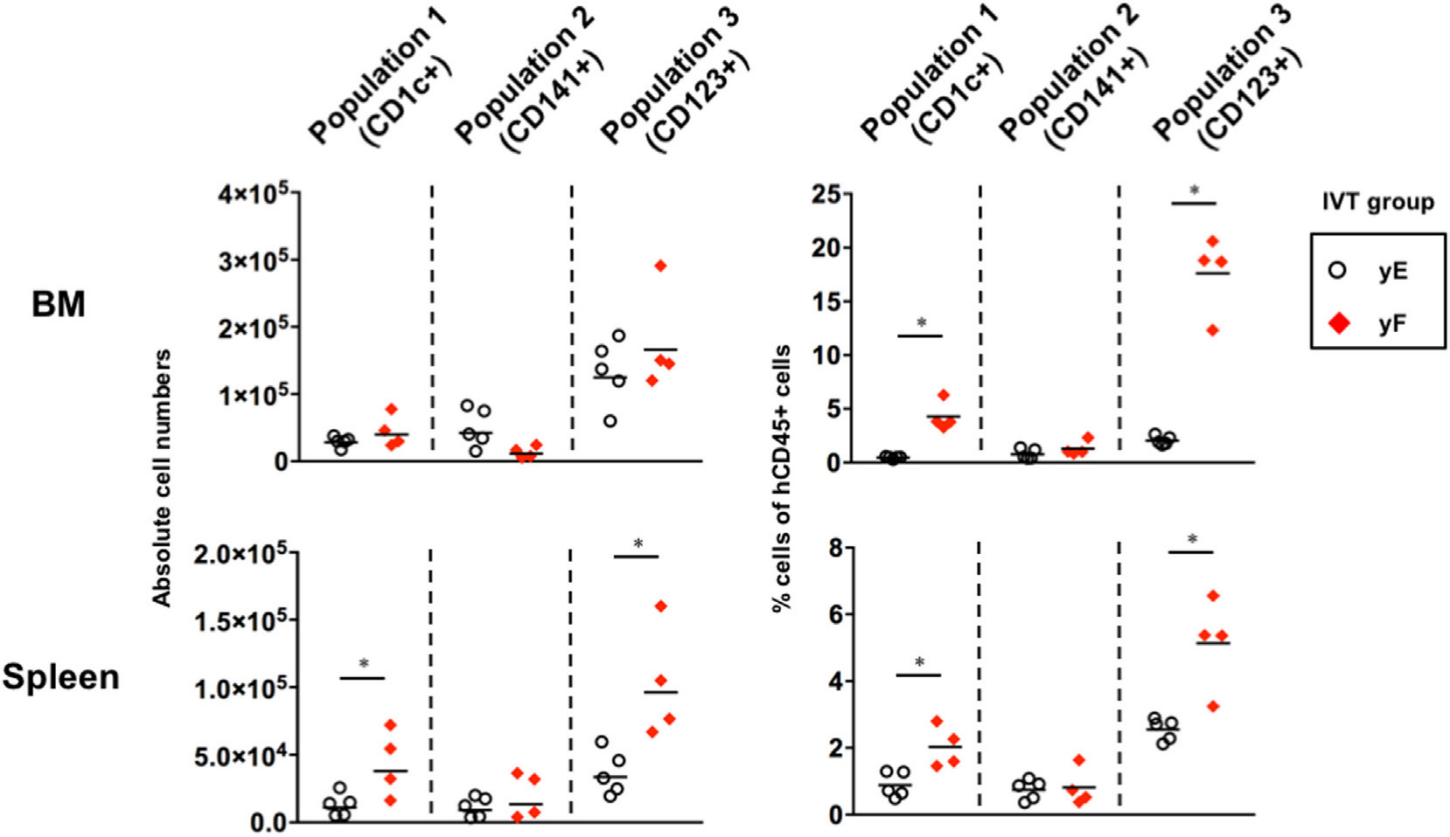
Effect of fms-related tyrosine kinase 3 ligand (Flt3-L) on the reconstitution of putative dendritic cell populations in the young hNOJ mice. Four-week-old hNOJ mice were subjected to in vivo transfection (IVT) with either the Flt3-L-expressing plasmid (Group yF) or the empty vector plasmid (Group yE). The absolute cell numbers (left panels) and the percentages (right panels) of CD1c^+^ population (Population 1), CD141^+^ population (Population 2), and CD123^+^ population (Population 3) in the bone marrow (BM) (upper panels) and spleen (lower panels) are shown. Data are the individual values (Group yE: *n* = 5, Group yF: *n* = 4) with the geometric means of the absolute cell numbers (left panels) or means of the percentages (right panels). Significant differences (*P < 0.05) were determined by the Mann–Whitney *U* test.

Whereas Ding et al. showed that treatment with Flt3-L contributes to robust expansion of pDCs as well as CD1c^+^ cDCs and CD141^+^ cDCs in the BM and spleen of humanized NOD/SCID mice (39), in our study, pDCs (Population 3) were not expanded by treatment with Flt3-L (Figure 4). Since Ding et al. treated mice with the cytokine earlier at 4 weeks after HSC transplantation (39), we evaluated the *in vivo* effect of Flt3-L in younger hNOJ mice. Four-week-old hNOJ mice were injected with either the Flt3-L-expressing plasmid (Group yF) or the empty vector (Group yE). Both pDCs (Population 3) and Population 1 significantly expanded in the BM and spleen in response to treatment with Flt3-L, while Population 2 did not (Figure 5). Interestingly, as shown in the previous experiment (Figure 4), an inverse pattern of expansion had been observed between CD141^+^ myeloid cells and pDCs. These results suggest that unknown age-related factors are involved in the differential developmental regulation of CD141^+^ cDCs and pDCs.

### Comparison of BM Hematopoietic Progenitor Populations Between the Young and Old hNOJ Mice

**Figure 6:**
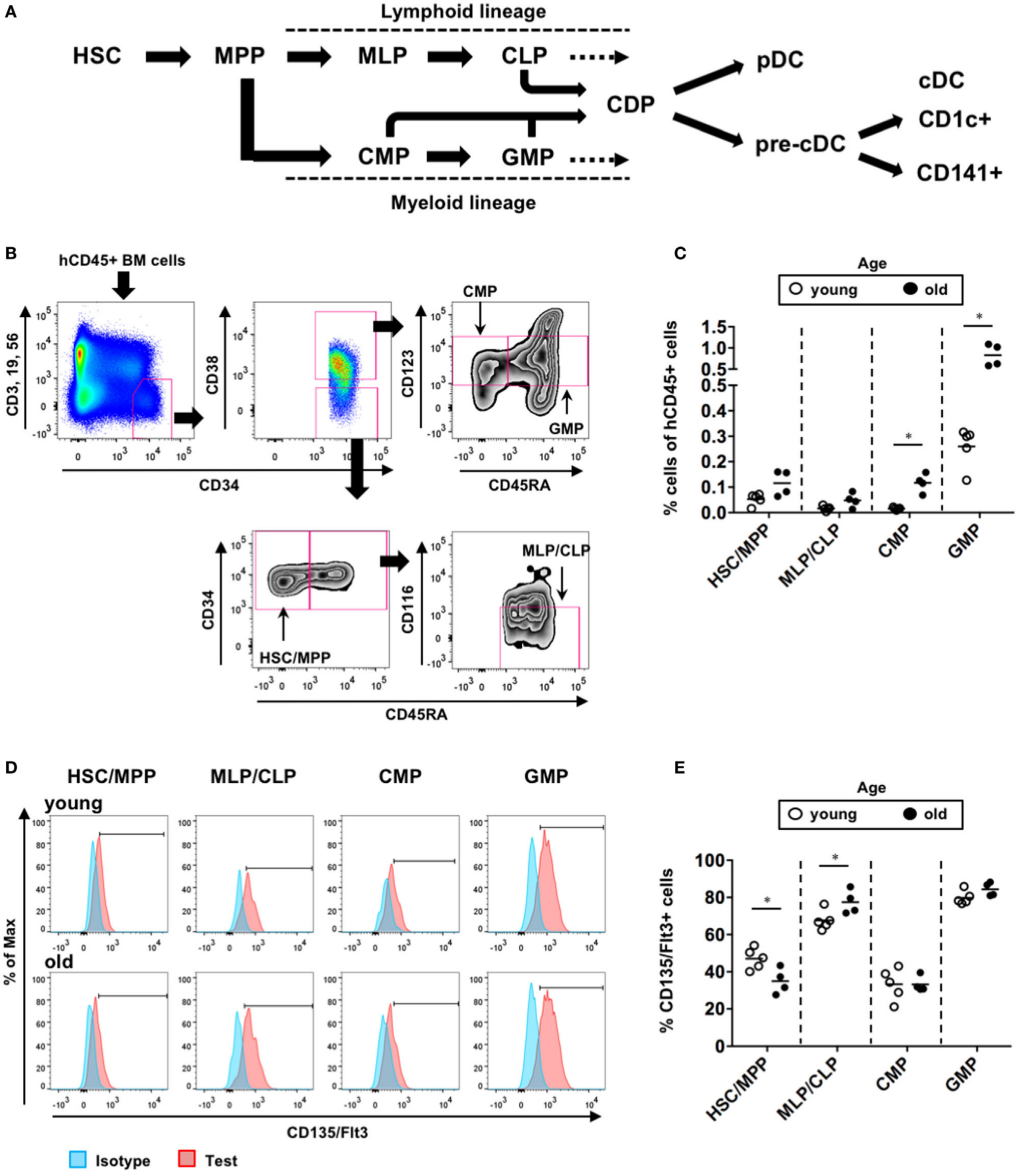
Composition of hematopoietic progenitors in hNOJ mice. The young (4-week-old) and old (16-week-old) hNOJ mice were subjected to *in vivo* transfection (IVT) with the empty vector plasmid. At 10 days post-IVT, bone marrow (BM) cells were analyzed by flow cytometry. **(A)** Schematic view of human dendritic cell hematopoiesis. **(B)** Identification of BM hematopoietic progenitors in hNOJ mice: hematopoietic stem cell (HSC)/MPP (CD34^+^CD38^−^CD45RA^−^), MLP/CLP (CD34^+^CD38^−^CD45RA^+^CD116^−^), CMP (CD34^+^CD38^+^CD45RA^−^CD123^lo^), and GMP (CD34^+^CD38^+^CD45RA^+^CD123^lo^). **(C)** Comparison of the frequency of each hematopoietic progenitor population between the young and old hNOJ mice. Data are the individual values (young: *n* = 5, old: *n* = 4). Significant differences (*P < 0.05) were determined by the Mann–Whitney *U* test. **(D)** Representative histograms of CD135/Flt3 and isotype staining. **(E)** Comparison of the frequency of CD135/Flt3^+^ cells within each hematopoietic progenitor population between the young and old hNOJ mice. Significant differences (*P < 0.05) were determined by an unpaired *t*-test.

We further investigated the populations of BM hematopoietic progenitors in the young and old hNOJ mice that were injected with the empty vector at 4 or 16 weeks of age, respectively. According to previous reports (41–43), hematopoietic progenitors within hCD45^+^CD34^+^ BM cells were divided into four populations in this study: CD38^−^CD45RA^−^ HSCs/multipotent progenitors (MPPs), CD38^−^CD45RA^+^CD116^−^ multi-lymphoid progenitors (MLPs)/common lymphoid progenitors (CLPs), CD38^+^CD45RA^−^CD123^lo^ common myeloid progenitors (CMPs), and CD38^+^CD45RA^+^CD123^lo^ granulocyte-macrophage progenitors (GMPs) (Figures 6A,B). When the frequencies of these populations were compared between the young and old hNOJ mice (Figure 6C), the old hNOJ mice showed higher frequencies of myeloid-lineage progenitors (CMPs and GMPs) than the young hNOJ mice. Although the old hNOJ mice tended to have a higher frequency of HSCs/MPPs than the young hNOJ mice, this difference was not significant. By contrast, there was a similar frequency of lymphoid-lineage progenitors (MLPs/CLPs) in the young and old hNOJ mice. Furthermore, when CD135/Flt3 expression on these hematopoietic progenitors was compared between the young and old hNOJ mice (Figures 6D,E), there was a higher frequency of CD135/Flt3^+^ HSCs/MPPs in the young hNOJ mice than in the old. By contrast, CD135/Flt3^+^ MLPs/CLPs were more abundant in the old hNOJ mice, while there were similar frequencies of CD135/Flt3^+^ CMPs and GMPs between the two groups. Although it remains unclear why the *in vivo* effect of Flt3-L differed according to age, these findings may partly explain the age-related differences in hNOJ mice with respect to the sensitivity to Flt3-L.

### Enhanced Maturation of Putative DC Populations Following Cytokine Induction

**Figure 7:**
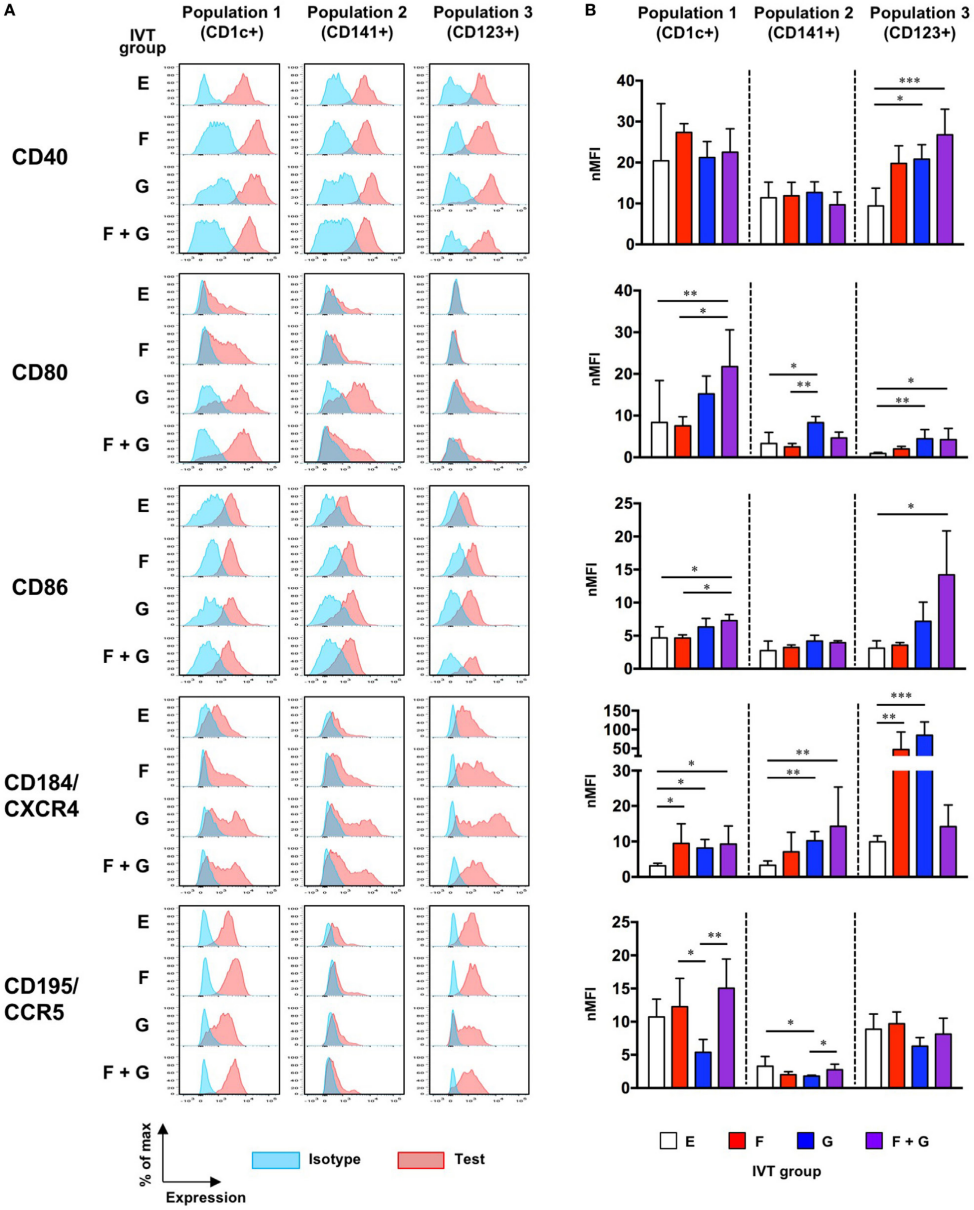
Maturation status of putative human dendritic cell populations in hNOJ mice following *in vivo* transfection (IVT). Splenocytes from each IVT group were subjected to flow cytometric analysis for maturation-associated markers. **(A)** Representative histograms of test marker and isotype staining. **(B)** Comparison of the normalized mean fluorescence intensity among IVT groups. Data are expressed as the mean ± SD (Group E: *n* = 9, Group F: *n* = 6, Group G: *n* = 5, Group F + G: *n* = 6). Significant differences (*P < 0.05, **P < 0.01, ***P < 0.001) were determined by the Kruskal–Wallis test followed by the Dunn’s multiple comparisons test.

We next examined whether treatment with cytokines affected the maturation status of each putative DC population in hNOJ mice. Since upregulation of CD40, CD80, CD86, and CD184/CXCR4 and downregulation of CD195/CCR5 are associated with DC maturation (44, 45), the expression of these markers was examined on splenocytes by flow cytometry. Because it was difficult to clearly distinguish positive and negative populations using some markers, and in some cases, background levels of fluorescence (staining with the isotype control) were different among the IVT groups (Figure 7A), the normalized mean fluorescence intensity (nMFI) was used as a quantitative measure of the expression of each marker (nMFI = test marker MFI/isotype MFI) (Figure 7B). Population-specific differences in the expression of maturation-associated markers were observed in the different IVT groups. Details are described below.

#### Population 1 (CD1c^+^)

CD40 was highly expressed even at the steady state (Group E), while the expression of CD184/CXCR4 was upregulated by cytokine treatment. The expression of CD80 was significantly upregulated by treatment with both Flt3-L and GM-CSF (Group F + G). Although the expression of CD86 was relatively low compared with CD80, a significant upregulation was also observed in Group F + G. CD195/CCR5, like CD40, was substantially expressed at the steady state (Group E), and downregulation was observed in response to treatment with GM-CSF alone (Group G).

#### Population 2 (CD141^+^)

The expression patterns of CD40 and CD184/CXCR4 in Population 2 were similar to those in Population 1. However, potent upregulation of CD80 was observed only after treatment with GM-CSF alone (Group G), and CD86 was not upregulated by any cytokine treatment. Furthermore, although CD195/CCR5 expression was lower at the steady state (Group E) than in Population 1 and Population 3, downregulation of CD195/CCR5 was observed in Group G, as with Population 1.

#### Population 3 (CD123^+^)

In contrast to Population 1 and Population 2, the expression of CD40 was upregulated by treatment with Flt3-L and/or GM-CSF. CD80 was hardly expressed at the steady state, but was also upregulated by GM-CSF alone (Group G) or in combination with Flt3-L (Group F + G). CD86 expression was significantly increased only after treatment with the combination of Flt3-L and GM-CSF (Group F + G). Whereas treatment with the combination of Flt3-L and GM-CSF upregulated the expression of CD40, CD80, and CD86, this was not the case for CD184/CXCR4 expression: upregulated expression of CD184/CXCR4 was observed only in response to single treatment with either Flt3-L (Group F) or GM-CSF (Group G). CD195/CCR5 was substantially expressed at the steady state. Although its expression might be downregulated in response to single treatment with GM-CSF (Group G), the level was not significant.

Collectively, our data demonstrate that the maturation of each population was enhanced by treatment with cytokines irrespective of the level of reconstitution. Specifically, combined treatment with Flt3-L and GM-CSF resulted in increased expression of the essential co-stimulatory molecules B7-1 (CD80) and B7-2 (CD86) by Population 1 and Population 3 in hNOJ mice. However, the influence of CD14^+^ monocyte-like cells that were substantially included in Population 1 and Population 2 should be reminded.

### Altered T Cell Subpopulations Following Cytokine Induction

**Figure 8:**
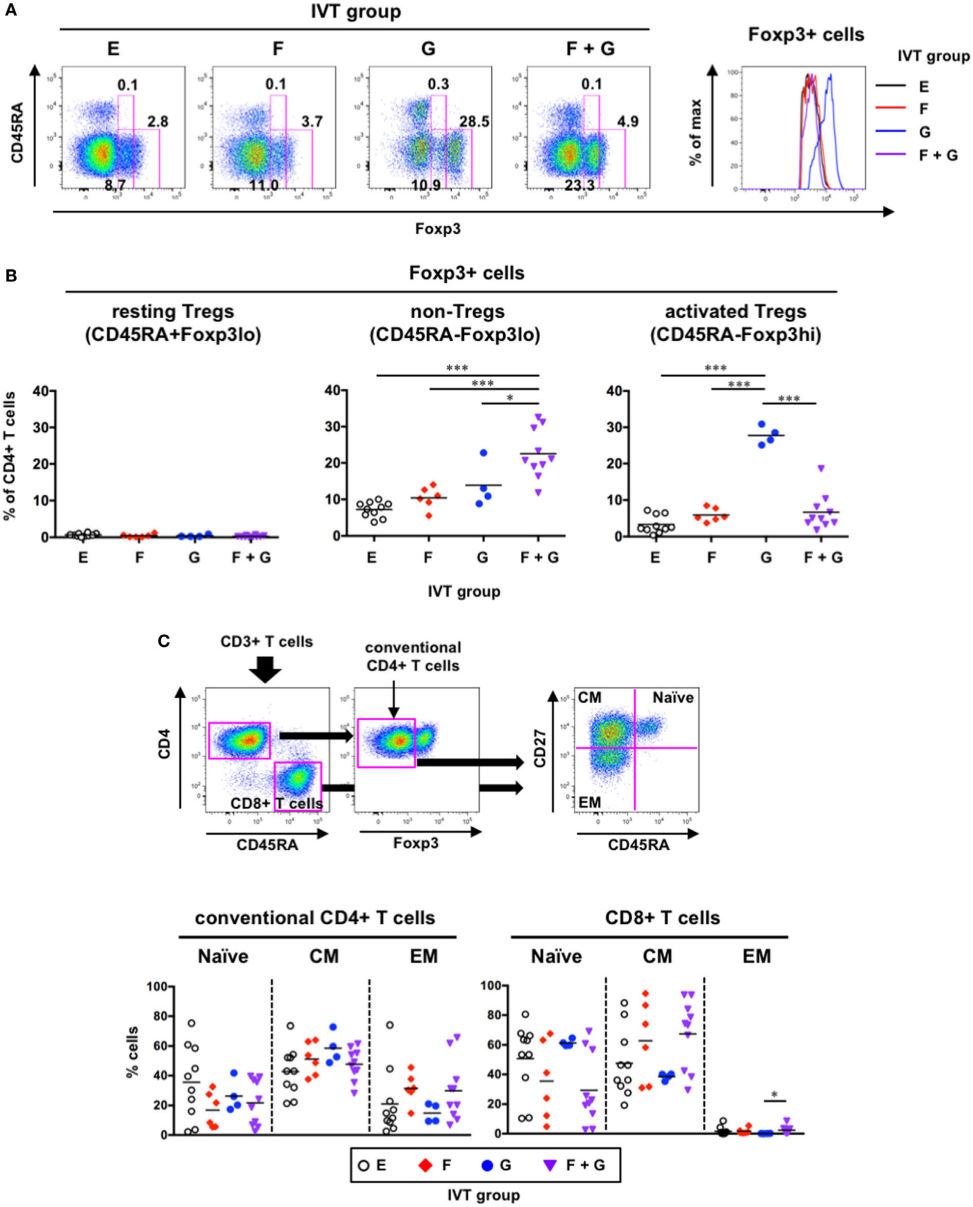
Phenotypic analysis of human T cells in hNOJ mice following *in vivo* transfection (IVT). Splenocytes from each IVT group were subjected to flow cytometric analysis for T cell phenotyping. **(A)** A representative gating strategy for resting regulatory T cells (Tregs) (CD45RA^+^Foxp3^lo^), non-Tregs (CD45RA^−^Foxp3^lo^), and activated Tregs (CD45RA^−^Foxp3^hi^) within Foxp3^+^CD4^+^ T cells. The histogram shows higher expression of Foxp3 in Group G. **(B)** Comparison of the percentages of Foxp3^+^CD4^+^ T cell subsets among the IVT groups. Significant differences (*P < 0.05, ***P < 0.001) were determined by one-way ANOVA followed by the Holm–Sidak’s multiple comparisons test (Group E: *n* = 10, Group F: *n* = 6, Group G: *n* = 4, Group F + G: *n* = 10). **(C)** A representative gating strategy for conventional CD4^+^ T cells and CD8^+^ T cells and differentiation stages of each T cell population. Significant difference (*P < 0.05) was determined by the Kruskal–Wallis test followed by the Dunn’s multiple comparisons test (Group E: *n* = 10, Group F: *n* = 6, Group G: *n* = 4, Group F + G: *n* = 10).

A humanized mouse model using the NSG-SGM3 strain, in which human stem cell factor, GM-CSF, and IL-3 are expressed, displayed not only increased reconstitution of human myeloid DCs but also skewed development of Foxp3^+^ Tregs (29). Therefore, we extended our flow cytometric analysis to the detection of Foxp3^+^CD4^+^ T cells in the spleen. We further subdivided Foxp3^+^CD4^+^ T cells into three subpopulations: resting Tregs (CD45RA^+^Foxp3^lo^), activated Tregs (CD45RA^−^Foxp3^hi^), and non-Tregs (CD45RA^−^Foxp3^lo^) (Figure 8A), as previously reported (46). Although the percentages of Foxp3^+^CD4^+^ T cells were increased in the presence of GM-CSF (Groups G and F + G), there were differences in the subpopulations of Foxp3^+^ cells within each group (Figures 8A,B). Whereas CD45RA^−^Foxp3^lo^ non-Tregs were only significantly increased in Group F + G (22.5 ± 6.7% of CD4^+^ T cells, Figure 8B, middle panel), CD45RA^−^Foxp3^hi^ activated Tregs were only significantly increased in Group G (27.8 ± 2.5% of CD4^+^ T cells, Figure 8B, right panel). CD45RA^+^Foxp3^lo^ resting Tregs were rarely observed in any of the IVT groups (Figure 8B, left panel).

Furthermore, to examine the differentiation status of the other T cell subsets (Foxp3^−^ conventional CD4^+^ T cells and CD8^+^ T cells), the T cells were divided into three subpopulations based on the expression patterns of CD45RA and CD27, as previously reported (47): naïve (CD45RA^+^CD27^+^), central memory (CM; CD45RA^−^CD27^+^), and effector memory (EM; CD45RA^−^CD27^−^) populations (Figure 8C, upper panels). There was a large amount of variability in the percentages of each subpopulation, and no significant changes were noted among the IVT groups (Figure 8C, lower panels). Nevertheless, CD8^+^ T cells consisted of substantial proportions of naïve and CM cells in all IVT groups (Figure 8C, lower right panel).

### Correlation Between the Maturation Status of Putative DC Populations and the Development of Foxp3^+^CD4^+^ T Cells Following Cytokine Induction

**Figure 9:**
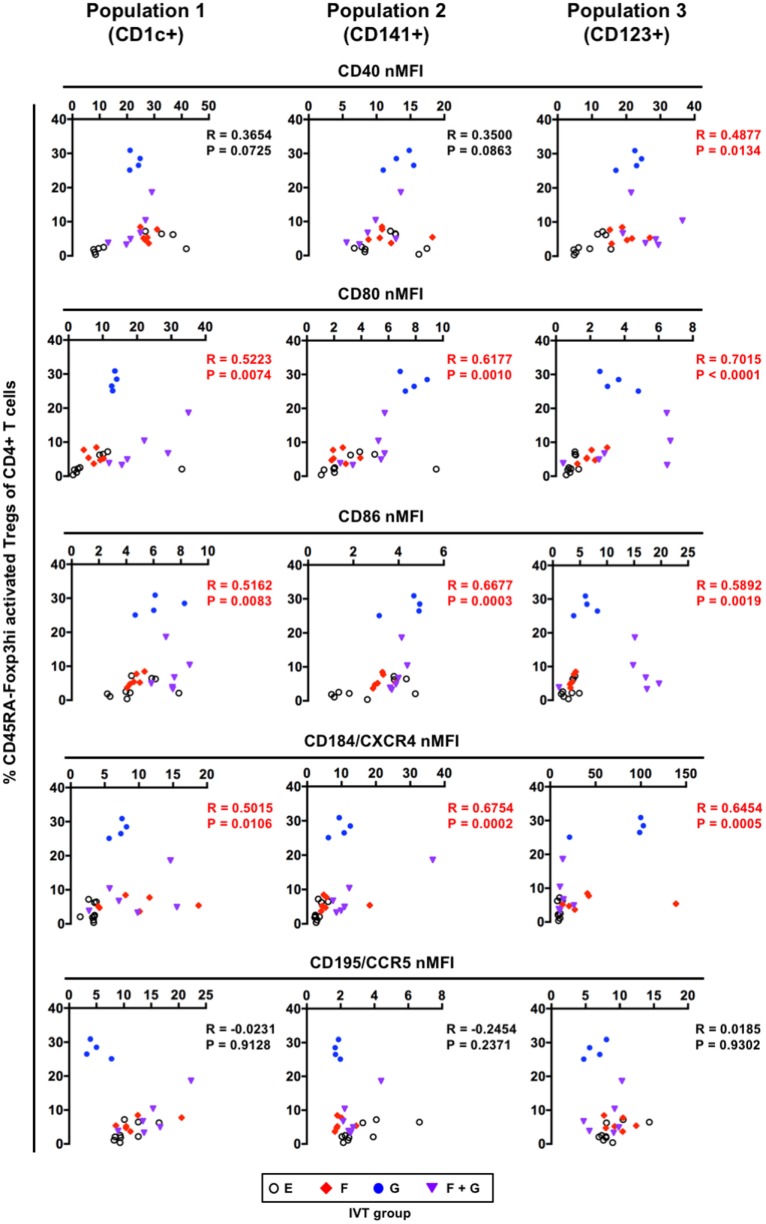
Correlation between the maturation status of putative dendritic cell populations and the development of CD45RA^−^Foxp3^hi^ activated regulatory T cells (Tregs). Individual normalized mean fluorescence intensity values for each maturation-associated marker in splenic CD1c^+^ population (Population 1), CD141^+^ population (Population 2), and CD123^+^ population (Population 3) (Figure 7B), and the percentages of CD45RA^−^Foxp3^hi^ activated Tregs (Figure 8B) were plotted (total *n* = 25, consisting of Group E: *n* = 9; Group F: *n* = 6; Group G: *n* = 4; Group F + G: *n* = 6). The Spearman’s rank correlation coefficient was used for statistical analysis.

Our data indicated that treatment with GM-CSF alone preferentially contributed to the enhanced development of CD45RA^−^Foxp3^hi^ activated Tregs in hNOJ mice (Figure 8B). However, GM-CSF alone had less impact on the reconstitution of putative DC populations including both CD14^−^ cDCs and CD14^+^ monocyte-like cells in the spleen (Figure 4). We, therefore, investigated the relationship between the maturation status of putative DC populations and the development of CD45RA^−^Foxp3^hi^ activated Tregs, and found that the expression of CD80 and CD86 as well as CD184/CXCR4 in every putative DC population positively correlated with the percentage of CD45RA^−^Foxp3^hi^ activated Tregs (Figure 9). These results indicate that the development of CD45RA^−^Foxp3^hi^ activated Tregs was associated with DC(-like cell) maturation, as characterized by the expression of co-stimulatory molecules (CD80 and CD86). Furthermore, when the percentages of CD45RA^−^Foxp3^lo^ non-Tregs were compared, a positive correlation was observed with the expression of some maturation-associated markers. However, it was only in Population 1 that the levels of CD80, CD86, and CD184/CXCR4 all correlated with the levels of Foxp3^+^ non-Tregs (Figure S3 in Supplementary Material), suggesting that the development of CD45RA^−^Foxp3^lo^ non-Tregs might be influenced by Population 1 rather than Population 2 and Population 3.

## Discussion

In this study, we asked how human Flt3-L and GM-CSF affected the reconstitution and maturation of DCs and the cellularity of T cells *in vivo* using hNOJ mice. We show that IVT by hydrodynamic injection-mediated gene delivery is a useful method to transiently introduce these cytokines into hNOJ mice. When comparing the concentrations of Flt3-L and GM-CSF induced by IVT, the Flt3-L concentration was much higher than the GM-CSF concentration even though the same expression vector (containing the same EF-1α promoter) was used to induce both cytokines. However, even the GM-CSF concentration in hNOJ mice was supraphysiological, since Flt3-L and GM-CSF levels are barely detectable in the circulation [Flt3-L: <100 pg/ml (48) and GM-CSF: <10 pg/ml (49)] in humans at the steady state. Furthermore, the Flt3-L and GM-CSF concentration was almost 100 times higher than in humanized NSG mice injected with Flt3-L DNA (31) or in humanized NSG-SGM3 mice in which GM-CSF is stably expressed (29). Although both the Flt3-L and GM-CSF concentrations were substantially decreased at 10 days post-IVT, we conducted *ex vivo* analyses of DCs and T cells at that time because the cytokine levels were still detectable and because the experiments by Chen et al. were conducted at 9 days post-injection of plasmids, when the concentrations of the introduced cytokines were also substantially decreased (31).

In hNOJ mice, three human DC subsets (CD1c^+^ cDCs, CD141^+^ cDCs, and pDCs) were reconstituted, as observed in other humanized mice (20, 50). Further phenotyping on the basis of the expression of HLA-DR, CD11c, CD303, CD4, and CD11b confirmed that all of these DC subsets were phenotypically similar to their equivalents in human blood. However, it should be noted that the pDC population might include pre-cDCs, since pre-cDCs and pDCs have similar expression of CD33 and CD123, as reported recently by See et al. (51), though this pDC population was not increased in the spleen when CD1c^+^ cDCs or CD141^+^ cDCs were increased. In addition to these DC subsets, we found that CD14^+^ monocyte-like cells substantially expressed CD1c and/or CD141 even at the steady state and that they were certainly increased after treatment with both Flt3-L and GM-CSF. The level of CD14 expression by CD14^+^ monocyte-like cells was low or intermediate, suggesting a similar phenotype of non-classical monocytes (40). By contrast, it is known that putative MoDCs such as CD14^+^ DCs and inflammatory DCs can express CD1c and CD141 (8, 34, 52). Interestingly, a recent single-cell RNA-seq analysis demonstrated that in human blood one of the CD1c^+^ DC subsets, “inflammatory” CD1c^+^ DCs (also designated CD1c^+^ B DCs), do not express CD14 on their cell surface but do express CD14 mRNA (53). Furthermore, in another humanized mouse model, it has been shown that CD1c^+^ cDCs in the BM contain both “non-inflammatory” (CD1c^+^ A) and “inflammatory” (CD1c^+^ B) DC subsets and that CD1c^+^ B DC-associated inflammatory markers including CD14 mRNA are upregulated after *in vivo* activation with TLR ligands, poly I:C, and/or R848 (50). Therefore, it is possible that cDCs, especially CD1c^+^ cDCs, could express CD14, depending on the tissue milieu. However, in this study, it was difficult to define whether CD14^+^ monocyte-like cells were categorized into monocyte, MoDC, or CD1c^+^ B DC populations. New technologies developed in recent years such as single-cell RNA-seq or CyTOF would be helpful for characterizing CD14^+^ monocyte-like cells in hNOJ mice.

Nevertheless, we show here the effect of *in vivo* expression of Flt3-L and GM-CSF on the reconstitution of CD1c^+^ cDCs, CD141^+^ cDCs, and pDCs. Although the effect on the DC reconstitution varied across the subsets and organs investigated, the introduction of both Flt3-L and GM-CSF reliably resulted in myeloid DC-rich hNOJ mice. On the other hand, pDCs failed to expand in the spleen in response to any of the cytokine treatments studied, despite the expected effect of Flt3-L on pDC reconstitution *in vivo* (12, 39) and despite the possible pre-cDCs within this population (51). Although it has been reported that GM-CSF impairs Flt3-L-induced pDC generation from BM progenitors in mice *in vitro* (54), pDCs failed to expand in hNOJ mice even in response to Flt3-L alone, indicating that this effect was independent of GM-CSF. However, pDCs did expand in the young hNOJ mice treated with Flt3-L alone though the possible contamination of pre-cDCs could not be excluded. These findings also suggest another possibility that the composition of hematopoietic progenitors might be affected by aging, i.e., by the amount of time after HSC transplantation. Human cDCs and pDCs arise independently of lineage commitment, in contrast to murine DC hematopoiesis (41, 55). With respect to myeloid-lineage progenitors (CMPs and GMPs), the frequency of CD135/Flt3^+^ cells was independent of aging in hNOJ mice. By contrast, higher frequencies of CD135/Flt3^+^ lymphoid progenitors (MLPs and CLPs) were detected in the old hNOJ mice, despite the fact that pDCs did not expand in response to Flt3-L treatment in these hNOJ mice. This might be in part due to the absence of human IL-3 in hNOJ mice, since IL-3 is required for the generation and survival of pDCs (56, 57). Interestingly, we found that when CD1c^+^ cDCs were expanded by treatment with certain cytokines, either CD141^+^ cDCs (or myeloid cells) or pDCs were expanded, but not both. However, these findings should be required close attention, since *in vitro* culture of human CD34^+^ HSCs with Flt3-L generates CLEC9A^+^ DCs, but they lack CD141 expression (58). Since it has been demonstrated recently that CLEC9A is a perfect discriminative surface marker for cDC1 (53), this marker should be helpful in the future study. Although the developmental regulation of each DC subset by Flt3-L and GM-CSF in hNOJ mice remains unclear, age-related unknown factors might be involved in the underlying mechanisms.

Dendritic cells play an essential role in the induction of not only immunity but also tolerance, and the maturation of DCs is considered to be crucial for the induction of T cell immunity (1). However, it has been suggested that treatment with Flt3-L alone may not be sufficient to generate fully functional DCs (10). Indeed, this study demonstrated that the effect of Flt3-L alone on the maturation in hNOJ mice was limited to, for example, CD184/CXCR4 upregulation on CD1c^+^ myeloid cells and pDCs. By contrast, GM-CSF alone or in combination with Flt3-L upregulated the expression of CD80, one of the co-stimulatory molecules, in all DC(-like) subsets. Strikingly, splenic Foxp3^+^CD4^+^ T cells preferentially expanded in hNOJ mice in the presence of GM-CSF. This finding is in agreement with earlier studies in mouse models: humanized NSG-SGM3 mice, in which human GM-CSF is stably expressed, showed skewed development of human Foxp3^+^ Tregs (29), and NOD mice, an animal model for type 1 diabetes, showed expansion of mouse Foxp3^+^ Tregs after treatment with mouse GM-CSF but not with mouse Flt3-L (59).

Human Foxp3^+^CD4^+^ T cells can be subdivided into three subpopulations on the basis of the expression of CD45RA and Foxp3: CD45RA^+^Foxp3^lo^ resting Tregs, CD45RA^−^Foxp3^hi^ activated Tregs, and CD45RA^−^Foxp3^lo^ non-Tregs (46). Whereas CD45RA^+^Foxp3^lo^ resting Tregs and CD45RA^−^Foxp3^hi^ activated Tregs are suppressive, CD45RA^−^Foxp3^lo^ non-Tregs are not suppressive, and are the highest producers of IL-17 among whole CD4^+^ T cells, suggestive of a T helper (Th)17 phenotype (46). Foxp3^+^CD4^+^ T cells in hNOJ mice consisted primarily of CD45RA^−^Foxp3^hi^ activated Tregs and CD45RA^−^Foxp3^lo^ non-Tregs. Remarkably, the developmental regulation of the two subpopulations differed, as CD45RA^−^Foxp3^hi^ activated Tregs were only increased after treatment with GM-CSF alone, whereas CD45RA^−^Foxp3^lo^ non-Tregs were only increased after treatment with both Flt3-L and GM-CSF. This differential regulation by Flt3-L has not been addressed elsewhere. Because the increased Foxp3^+^CD4^+^ T cells in hNOJ mice were distinguishable from naturally occurring Tregs, which have a CD45RA^+^ naïve phenotype (46, 60, 61), these cells had not recently migrated from the thymus but presumably had expanded in the spleen after interacting with antigen-presenting cells. It is not likely that GM-CSF acted directly on the T cells, since T cells in humanized NSG mice (29) and in humans (62) do not express the receptor for GM-CSF. It has been suggested that DCs, especially those expressing MHC II and co-stimulatory molecules (CD80 and CD86), play a major role in the development of Tregs (1, 63, 64). Our correlation analysis demonstrated that the development of CD45RA^−^Foxp3^hi^ activated Tregs was associated with the maturation status of all putative DC populations, particularly with respect to the expression of the B7 family co-stimulatory molecules (CD80 and CD86) by all putative DC populations. Interestingly, GM-CSF-treated human CD1c^+^ cDCs (65) and their equivalents in mice (66) can induce Tregs, suggesting a unique role for GM-CSF in the modulation of CD1c^+^ cDCs. Although whether GM-CSF can induce tolerogenicity in CD141^+^ cDCs and pDCs remains unknown, both DC subsets can induce Tregs under certain conditions (67, 68). Further characterization regarding tolerogenicity and which subsets of DCs are directly involved in the skewed development of CD45RA^−^Foxp3^hi^ activated Tregs in hNOJ mice should be undertaken in the future.

A recent study by Minoda et al. showed that human CD1c^+^ cDCs and CD141^+^ cDCs reconstituted in humanized NSG-A2 mice, into which HLA-A2 is genetically introduced, are functionally equivalent to mouse CD11b^+^ cDCs that promote Th2 and Th17 responses and mouse CD8^+^ cDCs that promote Th1 and CD8^+^ T cell responses, respectively (50). Interestingly, our correlation analysis suggests that the development of CD45RA^−^Foxp3^lo^ non-Tregs is associated with CD1c^+^ myeloid cells, but not CD141^+^ myeloid cells in terms of CD80 and CD86 expressions. Chen et al. demonstrated that induction of GM-CSF and IL-4 in humanized NSG mice resulted in T cell activation and differentiation toward a CD45RA^−^ memory phenotype (28). In particular, these humanized mice could induce antigen-specific CD4^+^ T cell responses, including the secretion of IFN-γ and IL-4, following immunization with tetanus toxoid (28), suggesting that Th1 and Th2 development can occur in humanized mice under suitable conditions. Whether hNOJ mice could induce antigen-specific T cell responses as well as immunity or tolerance in the cytokine setting tested in this study remains to be investigated.

In conclusion, this study could provide a platform for understanding the development of human DCs and Tregs *in vivo*. Furthermore, this study sheds light on the methodology of using conventionally available second-generation immunodeficient mice expressing certain human cytokines *in vivo*. However, it should be noted that the induced cytokine concentrations are transient and unphysiological in this system. Further improvement could be achieved using human cytokine knock-in immunodeficient mice.

## Ethics Statement

Human umbilical cord blood was donated by the Japanese Red Cross Society Kanto-Koshinetsu Block Blood Center (Tokyo, Japan), Sugiura Women’s Clinic (Tokyo, Japan), and Fukuda Hospital (Kumamoto, Japan) after receiving written informed consent. The use of human umbilical cord blood was approved by the Medical Research Ethics Committee of the NIID for the use of human subjects (Tokyo, Japan) (protocol numbers 500 and 585). All mice were treated in accordance with the guidelines approved by the Institutional Animal Care and Use Committee of the NIID (protocol numbers 114035 and 215005).

## Author Contributions

Study design: KT. Data curation: RI, SI, MK-I, YT-Y, and KT. Acquisition of data: RI and KT. Analysis and interpretation of data: RI, SI, and KT. Validation: RI, SI, MK-I, HT, MA, YT-Y, and KT. Writing the original manuscript: RI and KT. Review and/or revision of the manuscript: MA and YT-Y.

## Conflict of Interest Statement

The authors declare that the research was conducted in the absence of any commercial or financial relationships that could be construed as a potential conflict of interest.
