# Supplementary material for: Introduction of Human Flt3-L and GM-CSF into Humanized Mice Enhances the Reconstitution and Maturation of Myeloid Dendritic Cells and the Development of Foxp3+CD4+ T Cells
Source: Front Immunol. 2018 May 28;9:1042. doi: 10.3389/fimmu.2018.01042 (PMC5985304; doi:10.3389/fimmu.2018.01042)
Supplement: Supplementary file 1 [file image_1.PDF]

Figure S1

Iwabuchi et al.

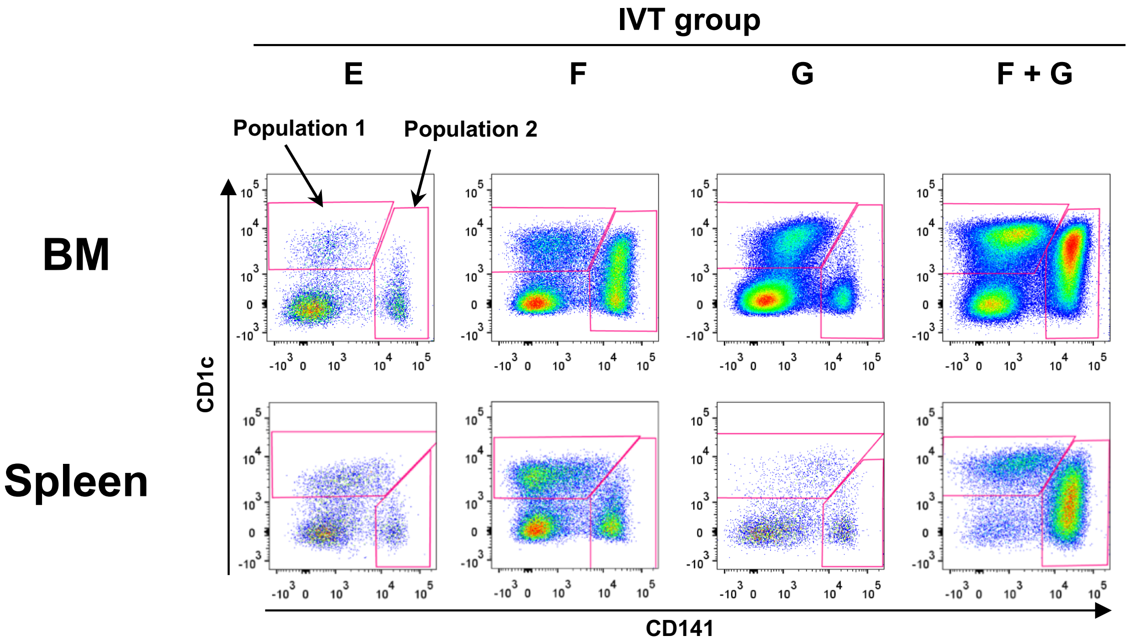

Figure S1. Representative pseudo-color plots of CD1c and CD141 expression on CD33⁺ myeloid cells in hNOJ mice following IVT.
CD1c and CD141 double-positive cells were induced mainly in the presence of Flt3-L, and these cells were included in CD141⁺ Population 2.
