# Supplementary material for: Introduction of Human Flt3-L and GM-CSF into Humanized Mice Enhances the Reconstitution and Maturation of Myeloid Dendritic Cells and the Development of Foxp3+CD4+ T Cells
Source: Front Immunol. 2018 May 28;9:1042. doi: 10.3389/fimmu.2018.01042 (PMC5985304; doi:10.3389/fimmu.2018.01042)
Supplement: Supplementary file 2 [file image_2.PDF]

Figure S2

Iwabuchi et al.

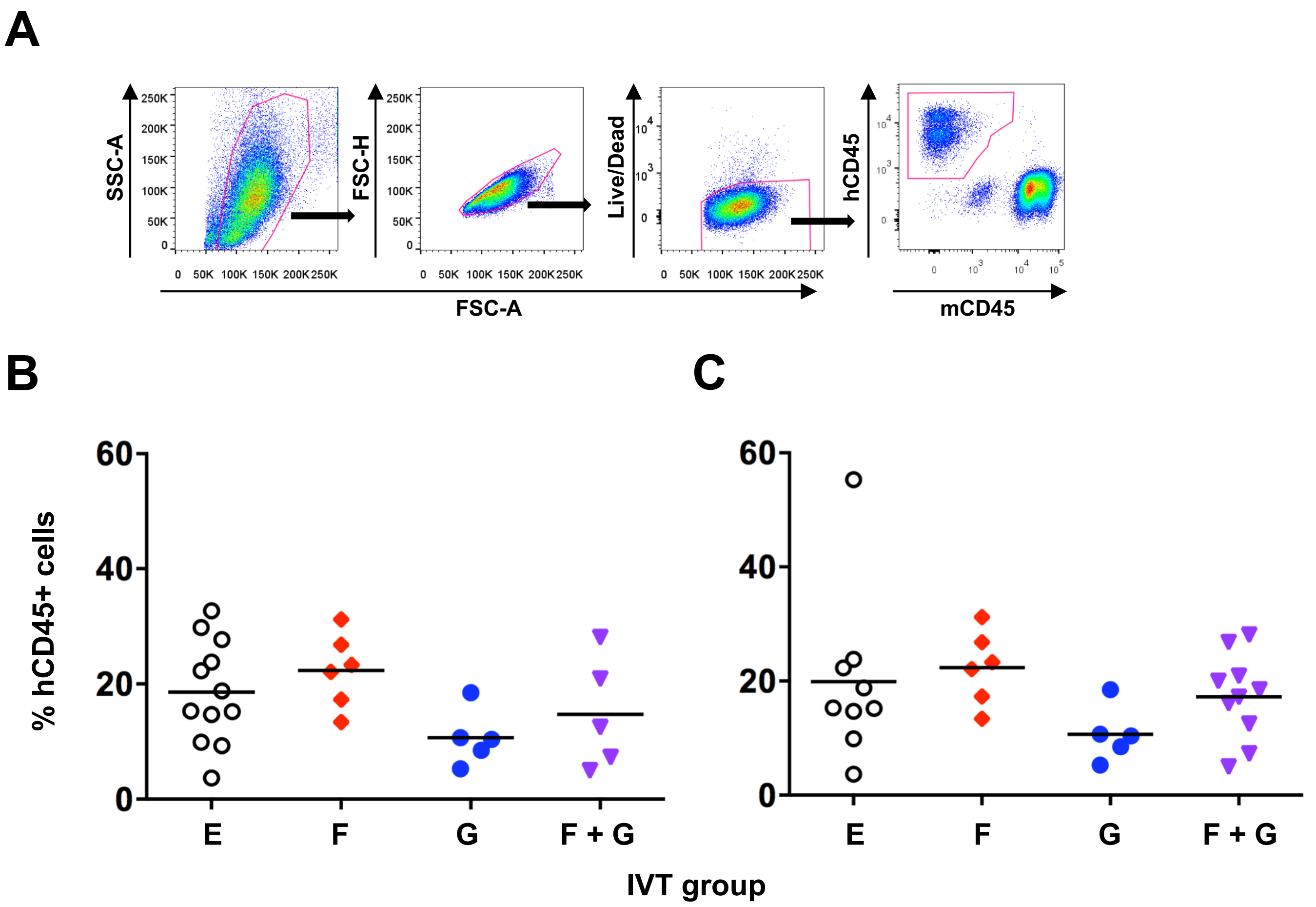

Figure S2. Chimerism in hNOJ mice used in Figure 4.
The percentage of hCD45⁺ cells within total peripheral blood cells at the initiation of IVT was compared across the IVT groups. **(A)** A representative gating strategy for flow cytometry of hCD45⁺ human leukocytes. **(B and C)** The absolute cell numbers in the BM **(B)** and spleen **(C)** from these mice are shown in Figure 4. The Kruskal-Wallis test followed by the Dunn's multiple comparisons test was used to compare groups, and no significant differences were observed.
