# Supplementary material for: Introduction of Human Flt3-L and GM-CSF into Humanized Mice Enhances the Reconstitution and Maturation of Myeloid Dendritic Cells and the Development of Foxp3+CD4+ T Cells
Source: Front Immunol. 2018 May 28;9:1042. doi: 10.3389/fimmu.2018.01042 (PMC5985304; doi:10.3389/fimmu.2018.01042)
Supplement: Supplementary file 3 [file image_3.PDF]

Figure S3

Iwabuchi et al.

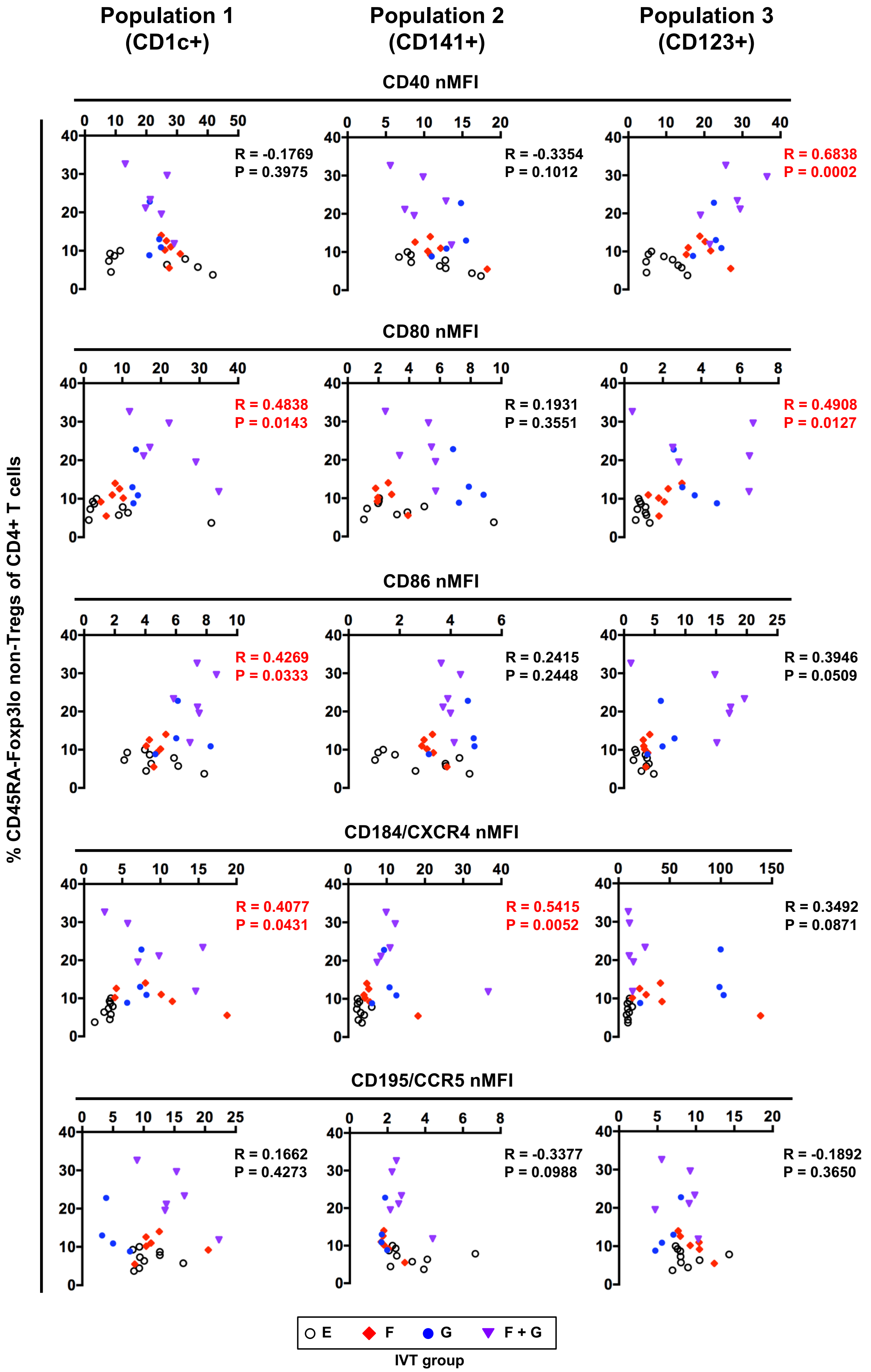

Figure S3. Correlation between the maturation status of putative DC populations and the development of CD45RA$^-$Foxp3^{lo} non-Tregs.

Individual nMFI values for each maturation-associated marker in splenic CD1c$^+$ Population 1, CD141$^+$ Population 2, and CD123$^+$ Population 3 (Figure 7B), and the percentages of Foxp3$^+$ non-Tregs (Figure 8B) were plotted (total: n = 25, consisting of Group E: n = 9; Group F: n = 6; Group G: n = 4; Group F + G: n = 6). The Spearman's rank correlation coefficient was used for statistical analysis.
