# Supplementary material for: Introduction of Human Flt3-L and GM-CSF into Humanized Mice Enhances the Reconstitution and Maturation of Myeloid Dendritic Cells and the Development of Foxp3+CD4+ T Cells
Source: Front Immunol. 2018 May 28;9:1042. doi: 10.3389/fimmu.2018.01042 (PMC5985304; doi:10.3389/fimmu.2018.01042)
Supplement: Supplementary file 4 [file table_1.PDF]

Table S1. List of hNOJ mice used in this study.

IVT* group	Mouse ID	Donor ID	Chimerism (%)**	Data presentation (Figure)
E	G330f1	607282	32.7	3A, 4A, S2B
E	G330f2	607282	27.7	3A, 4A, S2B
E	G330f3	607282	55.3	3A, 4B, 8A, 8B, 8C, S2C
E	G330f4	607282	29.8	3A, 4A, S2B
E	G340f1	610052	15.3	3A, 4A, 4B, 7A, 7B, 8B, 8C, 9, S2B, S2C, S3
E	G341f1	610051	22.3	2C, 3A, 3B, 4A, 4B, 7B, 8B, 8C, 9, S1, S2B, S2C, S3
E	G341f3	610051	15.2	2C, 3A, 4A, 4B, 7A, 7B, 8B, 8C, 9, S2B, S2C, S3
E	G341m2	610051	9.9	2A, 2C, 3A, 4A, 4B, 7B, 8B, 8C, 9, S1, S2B, S2C, S3
E	G341m6	610051	9.3	3A, 4A, 4B#, 7A, 7B, 8B, 8C, 9, S2B, S3
E	G376f3	703283	14.7	3A, 3B, 4A, 4B, 6C, 6E, 7A, 7B, 8B, 8C, 9, S2B, S2C, S3
E	G376m2	703283	3.7	3A, 4A, 4B, 6C, 6E, 7A, 7B, 8B, 8C, 9, S2B, S2C, S3
E	G376m3	703283	18.8	3A, 4A, 4B, 6C, 6D, 6E, 7A, 7B, 8B, 8C, 9, S2B, S2C, S3
E	G377f1	703284	23.8	3A, 4A, 4B, 6B, 6C, 6E, 7A, 7B, 8B, 8C, 9, S2B, S2C, S3
yE	G380m2	703281	12	5, 6C, 6D, 6E
yE	G380m4	703281	8.5	5, 6C, 6E
yE	G381f1	703282	5.3	5, 6C, 6E
yE	G386f1	501283	6.4	5, 6C, 6D, 6E
yE	G386m1	501283	4.1	5, 6C, 6D, 6E
Naïve	G328f1	607131	22.26	2B
Naïve	G329f1	607132	23.32	2B
Naïve	G329m1	607132	12.8	2B
Naïve	G329m2	607132	8.702	2B
F	G361f1	703241	23.3	1, 3A, 3B, 4A, 4B, 7A, 7B, 8B, 8C, 9, S1, S2B, S2C, S3
F	G361f3	703241	31.2	1, 3A, 3B, 4A, 4B, 7A, 7B, 8A, 8B, 8C, 9, S1, S2B, S2C, S3
F	G361m1	703241	13.4	1, 3A, 4A, 4B, 7B, 8B, 8C, 9, S2B, S2C, S3
F	G362f1	703271	26.8	1, 3A, 4A, 4B, 7A, 7B, 8B, 8C, 9, S2B, S2C, S3
F	G363f1	703271	22.1	1, 3A, 4A, 4B, 7A, 7B, 8B, 8C, 9, S2B, S2C, S3
F	G363m1	703271	17.3	1, 3A, 4A, 4B, 7A, 7B, 8B, 8C, 9, S2B, S2C, S3
yF	G380 f-1	703281	4.1	5
yF	G380 m-1	703281	12.8	5
yF	G380 m-3	703281	8.9	5
yF	G381 f-2	703282	4.4	5
G	G341f2	610051	18.5	1, 3A, 4A, 4B, 7A, 7B, 8B, 8C, 9, S1, S2B, S2C, S3
G	G341m3	610051	5.3	1, 3A, 3B, 4A, 4B, 7A, 7B, S1, S2B, S2C
G	G341m5	610051	10.7	1, 3A, 4A, 4B, 7A, 7B, 8A, 8B, 8C, 9, S2B, S2C, S3
G	G343m2	610131	10.4	1, 3A, 4A, 4B, 7A, 7B, 8B, 8C, 9, S2B, S2C, S3
G	G343m3	610131	8.5	1, 3A, 3B, 4A, 4B, 7A, 7B, 8B, 8C, 9, S2B, S2C, S3
F + G	G315f3	604124	20.0	1, 3A, 4B, 8B, 8C, S2C
F + G	G315f4	604124	17.2	1, 3A, 4B, 8B, 8C, S2C
F + G	G315f5	604124	28.1	1, 3A, 4A, 4B, 8B, 8C, S2B, S2C
F + G	G315f6	604124	26.8	1, 3A, 4B, 8B, 8C, S2C
F + G	G327f1	606232	20.9	1, 3A, 4A, 4B, 7A, 7B, 8B, 8C, 9, S1, S2B, S2C, S3
F + G	G327f2	606232	5.0	1, 3A, 3B, 4A, 4B, 7A, 7B, 8B, 8C, 9, S2B, S2C, S3
F + G	G327m1	606232	16.1	1, 3A, 4B, 7B, 8B, 8C, 9, S2C, S3
F + G	G327m2	606232	18.5	1, 3A, 3B, 4B, 7A, 7B, 8B, 8C, 9, S2C, S3
F + G	G337m1	608312	12.5	1, 3A, 4A, 4B, 7A, 7B, 8A, 8B, 8C, 9, S1, S2A, S2B, S2C, S3
F + G	G337m2	608312	7.3	1, 3A, 4A, 4B, 7A, 7B, 8B, 8C, 9, S2B, S2C, S3, S4A

* in vivo transfection (E: empty, yE: young E, F: Flt3-L, G: GM-CSF, yF: young F, F + G: Flt3-L + GM-CSF

** % hCD45+ cells within total peripheral blood cells

[#] Frequency only
